# Crystallizations of constructs: Lessons learned from a literature review

**DOI:** 10.1007/s40037-018-0422-0

**Published:** 2018-04-27

**Authors:** Meredith E. Young

**Affiliations:** Department of Medicine and Centre for Medical Education, Faculty of Medicine, McGill University, Montreal, Quebec Canada

**Keywords:** Review, Clinical reasoning, Scoping, Theory

## The story

Returning back to work following maternity leave, I was feeling a bit out of touch with the current literature on clinical reasoning, a literature with which I am generally quite familiar. I teamed up with a few colleagues, and drafted a proposal to synthesize the literature on the operationalization of clinical reasoning: If someone has claimed to measure, document, observe or demonstrate the presence or quality of clinical reasoning, what did they report as ‘data’? To our pleasant surprise, the project was funded, and we embarked on designing the search strategies, planning which databases to query, and taking steps to ensure we were engaging in high-quality synthesis work. We were lucky to have a strong academic librarian on our team, who invested time in helping me learn the ins and outs of a ‘rigorous’ review methodology. We did multiple iterations of the search strategy, we ran pilot searches for feasibility, and we ‘tested’ the database of articles generated by our search strategy.

Once you have devised a search strategy, there are different ways to test its quality. You can ask peers to review the strategy itself [1], or you can generate a list of articles that you think a good search should capture—be they seminal works of scholarship, works that have been written by particular expert authors, or articles that you know have captured the essence of the literature you are scouring. If the initial list of generated articles does not include this collection of key works, the search is adapted and strengthened following a careful analysis of indexing patterns and keywords (different approaches for adapting search strategies as seen, for example, in Hausner et al. [2, 3]).

Eager to ground our synthesis in a solid strategy, we independently generated a list of key citations and key authors we expected our search to detect. The expected players were there, and we were careful to include authors with different perspectives on clinical reasoning, and different approaches to data collection and analysis (e. g. experimentalists, qualitative researchers, assessment perspectives). In a moment of hubris, I put my own name on the list—I have published work on clinical reasoning, and thought that including myself amongst this illustrious group of prolific authors who have founded the field would be a more ‘sensitive’ way to test our search strategy. A comprehensive search of the literature on clinical reasoning should, after all, pick up my work—shouldn’t it?

## Surprising outcomes

The unsurprising outcome is that I found representation from those that you would expect—those who write often on clinical reasoning were, indeed, included in our pilot body of literature. What surprised me was that none of my work was captured by the search strategy. This did not bother my ego, but rather gave me pause—I’ve published articles on clinical reasoning, I’ve ‘measured’ diagnostic accuracy, I’ve carefully crafted experimental studies—I should be in there.

I immediately booked a meeting with our academic librarian to work through the findings of our literature probe. In preparing for the meeting, I pulled out the papers I have written that I thought should have been captured by the search strategy. That is when the problem started to crystallize—I realized that when I talk casually about my research, I describe my work using the phrase *clinical reasoning,* but I don’t use that phrase anywhere within my published work.

In presentations and discussions, I rely on the term *clinical reasoning* to describe what I do—‘I study the basic science of *clinical reasoning*’ or ‘I apply models and theories from cognition to understand *clinical reasoning’.* The term allows me to quickly explain my broad area of interest to others, to situate myself within a common ground for conversation, and to confer some value and relevance to my work. If it is a term helpful for conferring relevance, for supporting easy communication, and for bridging the basic science and applied context gap, why, then, have I not mobilized it in my manuscripts?

## Lessons learned

For the review itself, we have broadened the scope of our search and refined some methodological steps in order to deal with the complexity, or at least apparent vagueness, of the construct of clinical reasoning. From a pragmatic perspective, a lesson learned from this story is to include an academic librarian on your literature review team—the contribution of his/her expertise, knowledge, and wisdom cannot be overestimated.

But why the difference in terminology when I talk versus when I write? On reflection, I’ve noticed that in articles intended for peer review, I tend to use language that precisely describes what was measured or studied. Hence, I use phrases like *diagnostic accuracy, diagnostic decisions, symptom interpretation*, or *response time*. For me, this linguistic precision gives shape to an otherwise amorphous construct, serving to crystallize the concept of clinical reasoning into an outcome that I am able to measure, document, or demonstrate.

When I devise a study, or at least design an experiment, I have to decide which facet in the broad concept of interest—in this instance, *clinical reasoning*—will be the focus of this study. For example, in the context of a given study, studying *clinical reasoning* might crystallize to ‘I will contrast familiarity of certain aspects of a diagnostic case (very familiar or not at all) and monitor the diagnostic probabilities assigned’. This crystallization is reflected in the specificity of the language I use to describe the construct of interest—here, I view diagnostic accuracy as one crystallized facet of clinical reasoning. Each facet of the construct brings with it certain inherent assumptions; assumptions for this crystallization include that clinical reasoning can be represented and measured through diagnostic accuracy, and that good reasoning leads to an accurate diagnosis. If, instead, my study focused on the role of patient input on management decisions as indicative of ‘good’ clinical reasoning, my observations would focus on a different aspect of the construct, and different assumptions about ‘good’ reasoning would be at play. Each time a construct is crystalized into an observable or study-able facet, it will highlight different notions of ‘good’ clinical reasoning [4], and bring with it different assumptions regarding the construct itself.

Writing destined for peer review likely contains more specific language that refers to a given crystallization of the broader construct, such as clinical reasoning. In our review of the conceptualizations of clinical reasoning, we identified a total of 110 terms used to refer to the construct of clinical reasoning [5]. This vast amount of terms referring to clinical reasoning likely reflects different theoretical frames, areas of focus, and different ways to crystallize the construct to render a complex and multidimensional concept into an object of study. By no means would I suggest that a unification of terminology is needed. While a harmonization of terms would ease the job of synthesizing such a diverse literature, I believe that these diverse crystallizations of clinical reasoning reflect the specificity intended by the authors—they are reporting the ways in which they have operationalized, described, observed, measured, or identified the presence of clinical reasoning, broadly defined.

To me, the most significant lesson learned from this experience has been that clinical reasoning is unlikely to be a single entity, but rather a concept that is crystalized as a multitude of interconnected facets and terms. Clinical reasoning is short hand for many different things—a term used to describe the ‘thinking that health practitioners do’ which takes on specificity, shape, and crystalizes through the active research, teaching, or assessment process. While different crystallizations of a construct imbue specificity, this specificity may come at a cost of being seen as relevant to, or of being seen as a legitimate manifestation of, the overarching concept.

The differences in terminology for an amorphous concept are likely present within other constructs in health professions education such as competence, professionalism, and feedback. While the notion that constructs can manifest differently across different works is not in itself problematic, I would suggest that care and caution is likely needed to ensure the clear communication of the constructs and of their crystallizations within our work in HPE.

## Moral of the story

The moral of this story for me is to carefully consider how and why we use the language we do when we do. The strengths of relying on a short-hand term like *clinical reasoning* include an easy means to enter a broader conversation, the ability to identify your work within a known area within health professions education, and likely eases conversations across clinical, methodological, and even epistemological boundaries. The weaknesses may be unclear communication, or the proliferation of poorly understood ‘god terms’ [6]. I would suggest that we should be careful not to conflate the value of the different of terminologies—specificity remains important for shaping and reporting of research findings, and generalism is vital for translation and application of findings within educational contexts.

## References

[1] McGowan J, Sampson M, Lefebvre C (2010). An evidence-based checklist for the peer review of electronic search strategies (PRESS EBC). Evid. Based Libr. Inf. Pract..

[2] Hausner E, Guddat C, Hermanns T, Lampert U, Waffenschmidt S (2016). Prospective comparison of search strategies for systematic reviews: an objective approach yielded higher sensitivity than a conceptual one. J Clin Epidemiol.

[3] Hausner E, Guddat C, Hermanns T, Lampert U, Waffenschmidt S (2015). Development of search strategies for systematic reviews: validation showed the noninferiority of the objective approach. J Clin Epidemiol.

[4] Young ME, Dory V, Lubarsky S, Thomas A. How different theories of clinical reasoning influence teaching and assessment. Acad Med. (accepted) 10.1097/ACM.0000000000002303 29847325

[5] Young ME, Thomas A, Lubarsky S (2017). Defining clinical reasoning: findings from a BEME scoping study.

[6] Lingard L (2009). What we see and don’t see when we look at ‘competence’: notes on a god term. Adv. Health. Sci. Educ. Theory. Pract..

